# A transdiagnostic neuroanatomical signature of psychiatric illness

**DOI:** 10.1038/s41386-018-0175-9

**Published:** 2018-08-08

**Authors:** Qiyong Gong, Cristina Scarpazza, Jing Dai, Manxi He, Xin Xu, Yan Shi, Baiwan Zhou, Sandra Vieira, Eamon McCrory, Yuan Ai, Cheng Yang, Feifei Zhang, Su Lui, Andrea Mechelli

**Affiliations:** 1 Huaxi MR Research Center (HMRRC), Department of Radiology, West China Hospital of Sichuan University, Chengdu, China; 2 Department of Psychoradiology, Chengdu Mental Health Center, Chengdu, China; 3 Department of Psychiatry, West China Hospital of Sichuan University, Chengdu, China; 4 Department of Psychosis Studies, Institute of Psychiatry, Psychology & Neuroscience, King’s College London, London, UK; 5 Division of Psychology and Language Sciences, University College London, London, UK

**Keywords:** Diagnostic markers, Psychosis, Translational research, Brain

## Abstract

Despite an increasing focus on transdiagnostic approaches to mental health, it remains unclear whether different diagnostic categories share a common neuronatomical basis. The current investigation sought to investigate whether a transdiagnostic set of structural alterations characterized schizophrenia, depression, post-traumatic stress disorder, and obsessive-compulsive disorder, and determine whether any such alterations reflected markers of psychiatric illness or pre-existing familial vulnerability. A total of 404 patients with a psychiatric diagnosis were recruited (psychosis, *n* = 129; unipolar depression, *n* = 92; post-traumatic stress disorder, *n* = 91; obsessive-compulsive disorder, *n* = 92) alongside *n* = 201 healthy controls and *n* = 20 unaffected first-degree relatives. We collected structural magnetic resonance imaging scans from each participant, and tested for transdiagnostic alterations using Voxel-based morphometry. Inferences were made at *p* < 0.05 after family-wise error correction for multiple comparisons. The four psychiatric groups relative to healthy controls were all characterized by significantly greater gray matter volume in the putamen (right: *z*-score: 5.97, *p*-value < 0.001; left: *z*-score: 4.97, *p*-value = 0.001); the volume of this region was positively correlated with severity of symptoms across groups (*r* = 0.313; *p* < 0.001). Putamen enlargement was also evident in unaffected relatives compared to healthy controls (right: *z*-score: 8.13, *p*-value < 0.001; left: *z*-score: 9.38, *p*-value < 0.001). Taken collectively, these findings indicate that increased putamen volume may reflect a transdiagnostic marker of familial vulnerability to psychopathology. This is consistent with emerging conceptualizations of psychiatric illness, in which each disorder is understood as a combination of diagnosis-specific features and a transdiagnostic factor reflecting general psychopathology.

## Introduction

Modern psychiatry is based on the principle that mental disorders are separate categories with distinct etiologies and clinical presentations. However, it is well known that several individuals show clear signs of general psychopathology but do not fit neatly within the boundaries of any diagnostic categories [1]. Furthermore, it has been recognized for several decades that comorbidity (the coexistence of two or more disorders) is the rule rather than the exception in mental health, with half of people who meet diagnostic criteria for one disorder also meeting criteria for at least one another disorder [2]. Within existing diagnostic systems, therefore, it is acknowledged that there are not clear-cut boundaries between the different categories. For example, in the Diagnostic Statistical Manual of Mental Disorders (DSM), “there is no assumption that each category of mental disorder is a completely discrete entity with absolute boundaries dividing it from other mental disorders” [3]. Consistent with this notion, an increasing number of genetic and epidemiological studies are pointing to large overlap between different psychiatric disorders. For example, similar sets of genes [4] and environmental risk factors [5] have been found to underlie a range of diagnostic categories, including schizophrenia, depression, obsessive-compulsive disorder (OCD), and post-traumatic stress disorder (PTSD). These findings have informed an emerging conceptualization of psychiatric illness, in which each disorder is best understood as a combination of diagnosis-specific as well as transdiagnostic features or mechanisms [6–9]. For example, in one influential model, this transdiagnostic factor—referred to as factor *p*—captures general psychopathology in a manner similar to how factor *g* of general intelligence measures performance across a wide range of seemingly disparate cognitive tasks [9]. Understanding the nature of vulnerabilities across psychiatric disorders may be important in informing the development of transdiagnostic interventions aimed a decreasing risk of psychopathology across disorders—a key direction for intervention research [10].

If a transdiagnostic factor underlies multiple psychiatric disorders, then one would expect a common neuroanatomical signature to characterize different diagnostic categories. Currently, the neuroimaging literature comprises hundreds of studies on the neuroanatomical basis of psychiatric disease. The vast majority of these, however, have compared groups of patients who met criteria for a specific disorder with control groups of healthy individuals. Such studies, while valuable in shedding light on specific psychiatric disorders preclude any test as to whether neuroanatomical alterations are in fact shared across traditional diagnostic categories. Furthermore, because the vast majority of studies did not include unaffected relatives, it has generally not been possible to establish whether the observed alterations reflected the development of psychiatric illness or pre-existing familial vulnerability.

Our current understanding as to whether common neuroanatomical features characterize different disorders is based on meta-analyses of studies that were carried out on individual disorders using different methodologies [11]. Consequently, at present, it is unclear whether different diagnostic categories have a common underlying neuroanatomical basis and whether any such marker reflects actual psychiatric illness or pre-existing familial vulnerability. A recent study investigate examined the neural basis of three psychiatric disorders (schizophrenia, bipolar disorder, and major depression). Critically, this study includes high percentages of medicated patients, ranging from 37% for major depression to 64% for schizophrenia [12]. This raises the possibility that at least some of the findings might be attributed to treatment effects. The aim of the present investigation was therefore to examine neuroanatomical alterations across medication-naive individuals suffering from four psychiatric disorders, including schizophrenia, depression, PTSD, and OCD. We recruited a total of 404 patients with a psychiatric diagnosis, including psychosis (*n* = 129), unipolar depression (*n* = 92), PTSD (*n* = 91), and OCD (*n* = 92) and 201 healthy volunteers. In addition, in order to elucidate whether the transdiagnostic alterations were a marker of current psychiatric illness or reflects pre-existing familial vulnerability, we also recruited 20 unaffected first-degree relatives of patients with first-episode psychosis (FEP). All participants were scanned using the same scanner and acquisition sequence, ensuring comparability of the data across multiple diagnostic categories. We hypothesized that a common transdiagnostic neuroanatomical alteration would characterize all four disorders compared to healthy controls (HCs). Furthermore, we expected that that the degree of structural alteration would be related to severity of symptoms within each illness. Finally, we hypothesized that alterations in similar brain regions would, at least in part, be evident in unaffected relatives and therefore reflect underlying familial vulnerability rather than simply a marker of current psychiatric illness.

## Materials and methods

### Participants

All participants were recruited as part of a larger cohort study of psychiatric illness in the Chinese population of Han nationality. Subsets of the data used here have been used in previous studies [13–16]. The present investigation was approved by the local research ethics committee and all participants provided written informed consent. Exclusion criteria applicable to all participants included: (i) history of drug or alcohol abuse; (ii) pregnancy; and (iii) any physical illness such as hepatitis, cardiovascular disease, or neurological disorder, as assessed by interview and review of medical records. Further information on each group is provided below.

### First-episode psychosis

In all, 129 patients with FEP were included (Table 1). Diagnosis of FEP and duration of illness were determined by the consensus of two experienced clinical psychiatrists using the Structured Interview for the DSM-IV Axis I Disorder, Patient Edition (SCID) [17]. Psychopathology was measured on the day of scanning using the Positive and Negative Syndrome Scale (PANSS) [18]. At the time of scanning, all patients were medication-naive; this was assessed by asking participants whether they had ever taken medication for mental health issues and examining their clinical records.

**Table 1:** Demographic and clinical characteristics of participants

	FEP (*n* = 129)	MDD (*n* = 92)	PTSD (*n* = 91)	OCD (*n* = 92)	HC (*n* = 122)
Age (±SEM)	25.00 (0.69)	34.96 (1.25)	42.73 (1.09)	29.97 (0.88)	29.61 (1.30)
Gender	55 M:74 F	43 M:49 F	29 M:62 F	59 M:33 F	78 M:123 F
Years of education (±SEM)	13.00 (0.27)	12.83 (0.60)	7.10 (0.33)	14.16 (0.32)	12.33 (0.29)
Symptom scores (±SEM)	PANSS positive: 24.50 (0.56) PANSS negative: 16.56 (0.69) PANSS general: 46.54 (0.83) PANSS total: 95.47 (1.74)	HAM-D: 23.06 (0.39)	PCL: 49.28 (1.08) CAPS: 58.06 (1.28)	Y-BOCS obsession: 12.94 (5.08) Y-BOCS compulsion: 8.42 (5.31) Y-BOCS total: 21.38 (0.57)	

*FEP* first-episode psychosis, *MDD* major depressive disorder, *PTSD* post-traumatic stress disorder, *OCD* obsessive-compulsive disorder, *HC* healthy control, *PANSS* Positive and Negative Syndrome Scale, *PCL* PTSD checklist, *CAPS* Clinician Administered PTSD Scale, *HAM-D* Hamilton Depression Rating Scale, *Y-BOCS* Yale-Brown Obsessive Compulsive Scale, *SEM* standard error of the mean, *M* males, *F* females

### Major depressive disorder

Ninety-two participants with major depressive disorder (MDD) were included (Table 1). Diagnosis of MDD was made with the SCID [17]. Severity of depression was quantified using the 17-item Hamilton Depression Rating Scale (HAM-D [19]). All participants had a total HAM-D score ≥ 18 on the day of testing. At the time of scanning, all patients were medication-naive; this was assessed by asking participants and examining their clinical records.

### Post-traumatic stress disorder

Ninety-one participants with PTSD were included (Table 1). These participants were survivors of the 2008 Sichuan earthquake who had all physically experienced the earthquake, personally witnessed death, serious injury, or the collapse of buildings, and not suffered any physical injury. All participants were assessed using the PTSD Checklist (PCL) [20] and met threshold criteria (PCL score ≥ 35) for diagnosis of PTSD on the day of scanning. Patients were also assessed using the Clinician Administered PTSD scale [21]. At the time of scanning, all patients were medication-naive; this was assessed by asking participants and examining their clinical records.

### Obsessive-compulsive disorder

Ninety-two participants with OCD were included (Table 1). Diagnosis of OCD and assessment of symptom severity were based on the Yale-Brown Obsessive-Compulsive Scale (Y-BOCS) and the clinician-rated Y-BOCS symptom checklist [22]. At the time of scanning, all patients were medication-naive; this was assessed by asking participants and examining their clinical records.

### Healthy controls

In all, 201 HC subjects were included (Table 1). All were recruited by poster advertisement, and screened using the non-patient edition of the SCID [17] to confirm the lifetime absence of psychiatric illnesses. In addition, these subjects were interviewed to exclude individuals with a known history of psychiatric illness in first-degree relatives.

### Unaffected relatives

To explore whether any transdiagnostic neuroanatomic alterations detected in the four diagnostic groups were a marker of current psychiatric illness or reflected pre-existing familial vulnerability, we also recruited 20 unaffected first-degree relatives of patients with a diagnosis of FEP. The absence of psychiatric illness in these participants was established using the non-patient edition of the SCID [17].

### Data acquisition

High-resolution T1-weighted images were acquired using a 3 T magnetic resonance imaging (MRI) system (EXCITE, General Electric, Milwaukee, WI, USA) with a volumetric three-dimensional spoiled gradient recall sequence (TR = 8.5 ms, TE = 3.4 ms, flip angle = 12°, and slice thickness = 1.0 mm) with an eight-channel phase array head coil. A field of view of 24 cm^2^ was used with an acquisition matrix comprising 256 readings of 128 phase-encoding steps, producing 156 contiguous coronal slices with slice thickness of 1.0 mm and in-plane resolution of 0.47 mm × 0.47 mm.

### Data analysis

The T_1_-weighted volumetric images were pre-processed using voxel-based morphometry as implemented in SPM12 software (http://www.fil.ion.ucl.ac.uk/spm) running under Matlab 7.1 (Math Works, Natick, MA, USA). In particular we used the Diffeomorphic Anatomical Registration using Exponentiated Lie algebra (DARTEL) SPM8 toolbox [23]; this involves the creation of a dataset-specific template and the segmentation of each individual image using such template, with the aim of maximizing accuracy and sensitivity [24]. The following steps were followed: (1) checking for scanner artifacts and gross anatomical abnormalities for each subject; (2) setting the image origin to the anterior commissure; (3) using the DARTEL toolbox to produce a high-dimensional normalization protocol; (4) checking for homogeneity across the sample; and (5) using standard smoothing (i.e. 8 mm). A “modulation step” was also included in the normalization in order to preserve the information about the absolute gray matter values [25]. After pre-processing, we obtained smoothed, modulated, normalized images for each of our four datasets. These were used to compare patients and controls using an analysis of covariance (ANCOVA), with age, gender, and total gray matter volume modeled as covariates of no interest. Neuroanatomical alterations common to the four diagnostic groups were identified by comparing FEP, PTSD, MDD, and OCD patients against HCs (e.g. FEP, PTSD, MDD, and OCD vs HC) and then using the inclusive masking option (at *p* < 0.05 uncorrected) in SPM12 software to identify those regions that survived the diagnosis-specific comparisons (i.e. FEP vs HC; PTSD vs HC; MDD vs HC; and OCD vs HC). This enabled us to detect alterations in the FEP group that reflect a transdiagnostic feature of psychiatric illness. Statistical inferences were made at *p* < 0.05 after family-wise error (FWE) correction with a minimum extent threshold of 10 voxels. Finally, in order to elucidate whether transdiagnostic alterations were a marker of current psychiatric illness or reflected pre-existing familial vulnerability, we performed statistical comparison between unaffected relatives and HCs, using a mask that comprised all voxels showing a transdiagnostic alteration in the ANCOVA. This mask was created using the Neuromorphometrics atlas (http://www.neuromorphometrics.com/) as implemented in SPM12. This statistical comparison was carried out using a two-sample *t*-test and, once again, statistical inferences were made at *p* < 0.05 after FWE correction with a minimum extent threshold of 10 voxels.

## Results

**Fig. 1:**
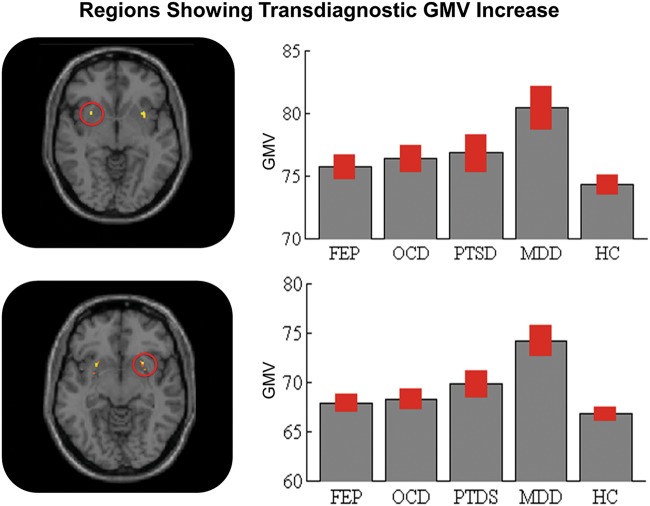
Transdiagnostic gray matter increases in patients relative to healthy controls. Left: mean gray matter volume in the five groups; the values on the *y*-axis refer to cubic millimeters per voxel with error bars representing SD. Right: regions of the left and right putamen where transdiagnostic increases were detected. FEP first-episode psychosis, OCD obsessive-compulsive disorder, PTSD post-traumatic stress disorder, MDD major depressive disorder, HC healthy controls, GMV gray matter volume measured as mm^3^ of gray matter per voxel

There were no regions with lower gray matter volume in patients relative to HCs across all diagnostic groups. However, the putamen showed higher gray matter volume in all four psychiatric groups relative to HC; this effect was evident both in the right (cluster = 32 voxels; *x* = 32 *y* = 6 *z* = −2; *z*-score: 5.97; *p*-value < 0.001 after FWE correction) and the left (cluster = 30 voxels; *x* = −30 *y* = 5 *z* = −7; *z*-score: 4.97; *p*-value = 0.001 after FWE correction) hemisphere (Fig. 1).

**Fig. 2:**
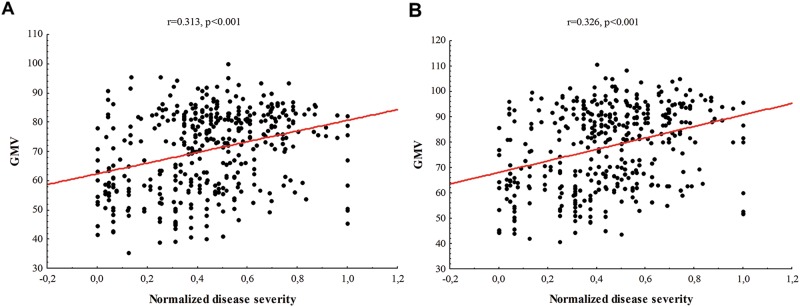
Partial correlation between gray matter volume in the right (**a**) and left (**b**) putamen and the disease severity, controlling for age and gender. To ensure disease severity scores were comparable across different disorders, these scores were normalized accordingly within each diagnostic group with the following formula: normalized individual value = (individual value − minimum score)/(maximum score − minimum score). Following the application of this formula, the disease severity score of each patient ranged between 0 and 1. GMV gray matter volume measured as mm^3^ of gray matter per voxel

Next, we considered whether the neuroanatomical increase in the bilateral putamen of patients relative to HCs was associated with severity of symptoms. This involved performing partial correlation analyses between gray matter volume in the left and right putamen and clinical scores within each diagnostic group, controlling for age and gender. This revealed significant positive correlations in the whole patient sample as well as in each individual diagnostic group (Table 2 and Fig. 2); in other words, higher gray matter volume in the putamen was associated with higher severity of symptoms.

**Table 2:** Association between gray matter volume in the bilateral putamen and severity of symptoms

	Right putamen		Left putamen	
Diagnosis	Coordinates	Partial correlation	Coordinates	Partial correlation
All patients	29 9 −8	*r* = 0.313, *p* < 0.001	−29 8 −5	*r* = 0.326, *p* < 0.001
FEP	28 7 8	*r* = 0.072, *p* = 0.731	−27 8 6	*r* = 0.178, *p* = 0.042
MDD	26 12 −8	*r* = 0.240, *p* = 0.023	−26 10 −5	*r* = 0.101, *p* = 0.512
PTSD	32 5 −9	*r* = 0.247, *p* = 0.020	−27 9 −8	*r* = 0.210, *p* = 0.050
OCD	24 11 0	*r* = 0.275, *p* = 0.009	−24 11 1	*r* = 0.253, *p* = 0.017

Symptoms were measured using PANSS total score for FEP; HAM-D score for MDD; CAPS score for PTSD; and Y-BOCS total score for OCD patients. The reported results refers to partial correlations, where age and gender were controlled in the correlation between GM and disease severity.

*FEP* first-episode psychosis, *MDD* major depressive disorder, *PTSD* post-traumatic stress disorder, *OCD* obsessive-compulsive disorder

**Fig. 3:**
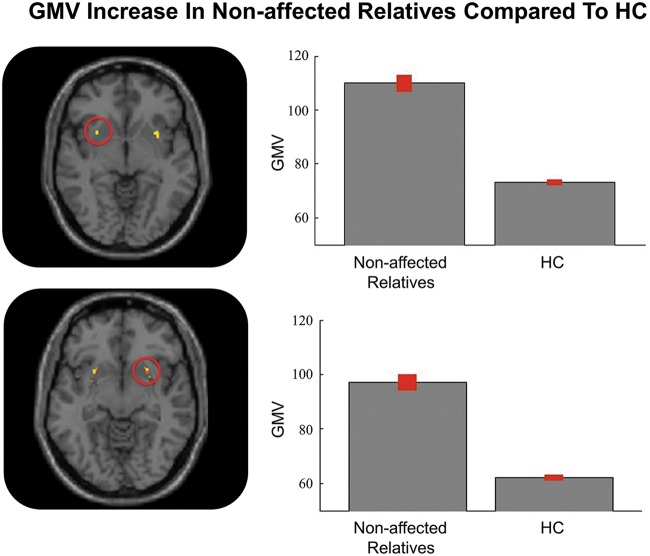
Transdiagnostic gray matter increases in unaffected relatives relative to healthy controls. Left: mean gray matter volume in the two groups; the values on the *y*-axis refer to cubic millimeters per voxel with error bars representing SD. Right: regions of the left and right putamen where transdiagnostic increases were detected. GMV gray matter volume measured as mm^3^ of gray matter per voxel

To elucidate whether the transdiagnostic increase in the bilateral putamen is a marker of current psychiatric illness or reflected pre-existing familial vulnerability, we performed a region of interest analysis in 20 unaffected first-degree relatives of patients with a diagnosis of FEP. This involved creating a mask of the bilateral putamen (total size: 62 voxels) and then performing a two-sample *t*-test to compare unaffected relatives and our group of HCs within this bilateral mask. The putamen showed higher gray matter volume in the group of unaffected relatives compared to HCs; this effect was evident both in the left (cluster = 30 voxels; *x* = −32 *y* = −8 *z* = 2; *z*-score: 9.39; *p*-value < 0.001 after FWE correction) and the right (cluster = 32 voxels; *x* = 32 *y* = −6 *z* = 2; *z*-score: 8.13; *p*-value < 0.001 after FWE correction) hemisphere Fig. 3.

## Discussion

The aim of this study was to test for common underlying neuroanatomic alterations across several diagnostic categories. This was achieved by investigating brain structure in medication-naive individuals with a number of psychiatric disorders, including schizophrenia, depression, OCD, and PTSD. Our findings indicated a transdiagnostic neuroanatomic signature across different diagnostic categories: an increase in gray matter volume of the bilateral putamen in all patient groups relative to HCs. The degree of increase was correlated with symptom severity and was also evident in unaffected first-degree relatives. Critically, as the individuals included in the current study were all medication-naive at the time of the scanning, the current results cannot be explained by medication effects.

Consistent with our first hypothesis of transdiagnostic neuroanatomical alterations, our investigation revealed higher gray matter volume of the putamen across all four disorders. The putamen receives afferent inputs from several cortical regions and via the thalamus sends neural projections back to the neocortex [26]. Through these cortico–striato–thalamo neural loops, the putamen serves as a center for the integration of high-level cognitive, motor, and limbic processes [27]. Given its role in these processes, it is unsurprising that dysregulation of this region is thought to underlie the emergence of cognitive and clinical symptoms in psychiatric disease [28]. Previous studies have reported higher gray matter volume in the putamen of patients relative to HCs in schizophrenia [29, 30] and OCD [31, 32]. As dopamine is a key neurotransmitter in the putamen and D2 dopamine receptors are a major target of typical antipsychotic medications, the observed increases have typically been interpreted as a consequence of D2 blockade induced by antipsychotic medication [33]. However, it is not possible to determine the functional significance of increased putamen volume observed in the present study. Future studies using healthy high-risk samples, which systematically map structural and functional indices within a longitudinal design, are necessary to shed light on what these observed structural differences may mean at a mechanistic level of disease progression and presentation.

Consistent with our second hypothesis of an association between transdiagnostic neuroanatomical alteration and severity of symptoms, gray matter volume in the putamen was positively correlated with higher severity of symptoms; this correlation was evident when considering each diagnostic group separately as well when combining the four disorders into a single sample (*n* = 404). A similar positive association between gray matter volume in the putamen and severity of symptoms had been reported in individuals with OCD [34] and PTSD [35]; however, to our knowledge, this is not a common finding in psychiatric neuroimaging, and as such requires replication. A previous investigation had reported a progressive loss of gray matter volume in the putamen in patients with schizophrenia with poor long-term outcome relative to those with good long-term outcome [36]. This had led to the suggestion that putamen enlargement may be a marker of responsiveness to antipsychotic treatment and even act as a protective factor against poor long-term outcome [37]. Our investigation extends this interpretation by suggesting that higher gray matter volume of the putamen is associated with a more severe clinical presentation across diagnostic boundaries. Because disease-specific clinical scales were used to measure severity of symptoms in each diagnostic group, higher gray matter volume of the putamen is likely to reflect generic symptomatology rather than the correlate of specific symptoms that are common to all four disorders.

To elucidate whether the transdiagnostic increase in the bilateral putamen is a marker of current psychiatric illness or reflected pre-existing familial vulnerability we performed a region of interest analysis in 20 unaffected first-degree relatives of patients with a diagnosis of FEP. Consistent with our third hypothesis, we detected a significant gray matter volume increase in the left and right putamen within this sample. This suggests that putamen enlargement is marker of underlying familial vulnerability rather than the disease itself. Here the term “familial” refers to effects that are shared between family members, without necessarily assuming a genetic or environmental etiology.

Taken collectively, our findings are consistent with emerging conceptualizations of psychiatric illness, in which each disorder is best understood as a combination of diagnosis-specific features and a transdiagnostic factor reflecting general psychopathology [6–9]. In particular, the observation of putamen enlargement in patients as well as unaffected relatives provides neurobiological evidence, which supports conceptualizations of mental illness based on cross-diagnostic vulnerability [38]. Given the role of the putamen in the integration of high-level cognitive, motor, and limbic processes, we speculate that disruption of these processes may represent a transdiagnostic feature of psychiatric disease. These processes could therefore be used as target in the development of novel transdiagnostic interventions aimed a decreasing risk for psychopathology across multiple disorders [10]. We note that the term “transdiagnostic” can be used to mean “across diagnoses” as well as “above and beyond diagnosis”. Here we use this term to refer to effects that are evident across different psychiatric disorders; in other words, we are not referring to the idea of abandoning current categorical categories to go “above and beyond” them (for interesting discussions on this topic [39, 40]).

A strength of the present investigation is that at the time of scanning, patients were experiencing their first episode of the illness and were still medication-naive. This means that the effects reported here cannot be explained by illness chronicity or medication—two common confounds in neuroimaging studies of psychiatric disease. A further strength is that all six groups were scanned using the same MRI scanner and image acquisition parameters over the same period of time, and therefore our results cannot be explained by systematic differences in the acquisition of the data. However, the present investigation has at least three limitations that warrant careful consideration. First, although we can exclude medication, age, and gender as potential confounds, there are other sources of neuroanatomical variability such as intelligence quotient and socioeconomic status that were not assessed and which may have contributed to the results. Second, because the data were acquired using a cross-sectional rather than a longitudinal design, it was not possible to unambiguously distinguish between correlates of vulnerability to psychiatric illness and correlates of psychiatric illness itself. However, the fact that we did detect a significant effect in unaffected relatives supports the notion that putamen enlargement is a marker of vulnerability rather than the illness itself. Third, the group of unaffected relatives (*n* = 20) was noticeably smaller than the other five groups. Nevertheless, we note that a group size of 20 is considered appropriate for a neuroimaging investigation [41], and that the bilateral effect detected in this group relative to HCs would have been statistically significant even if we had used a more conservative whole-brain FWE correction for multiple comparisons. Finally, while the aim of the present investigation was to examine common underlying neuroanatomic alterations across several diagnostic categories, it was not our intention to assume or imply that there are no disorder-specific correlates. We are therefore reporting disease-specific effects in the Supplementary Material for completeness (see Supplementary Material).

In conclusion, the present study has revealed putamen enlargement as a transdiagnostic marker of psychiatric illness; the observation of this difference in non-affected relatives suggests that this may reflect underlying familial vulnerability to general psychopathology. This raises the prospect that this region could be used to assess degree of familial vulnerability to psychopathology across traditional diagnostic boundaries, and thus adds to the development of psychoradiology (https://radiopaedia.org/articles/psychoradiology). These findings are consistent with emerging conceptualizations of psychiatric illness, in which each disorder is best understood as a combination of diagnosis-specific features and a transdiagnostic factor reflecting general psychopathology [38, 42].

## Electronic supplementary material

Supplementary Material
