# Supplementary material for: A transdiagnostic neuroanatomical signature of psychiatric illness
Source: Neuropsychopharmacology. 2018 Aug 8;44(5):869–75. doi: 10.1038/s41386-018-0175-9 (PMC6461829; doi:10.1038/s41386-018-0175-9)
Supplement: Supplementary file 1 — Supplementary Material [file 41386_2018_175_MOESM1_ESM.docx]

**Supplementary Material**

**A Transdiagnostic Neuroanatomical Signature of Psychiatric Illness**

Qiyong Gong^1,2,3^, MD, Ph; Cristina Scarpazza^4,5^, Psy, PhD; Jing Dai^2^, MD; Manxi He^2^, MD; Xin Xu^3^, MD; Yan Shi^3^, MS; Baiwan Zhou^1^, Sandra Vieira^4^, MD; Eamon McCrory^5^, Psy, Yuan Ai^1^, MD; Cheng Yang^1^, MD; Feifei Zhang^1^, MD; Su Lui^1^, MD, PhD; Andrea Mechelli^4^, PhD

^1^Huaxi MR Research Center (HMRRC), Departments of Radiology, West China Hospital of Sichuan University, Chengdu, China.

^2^Department of Psychoradiology, Chengdu Mental Health Center, Chengdu, China.

^3^Department of Psychiatry, West China Hospital of Sichuan University, Chengdu, China.

^4^ Department of Psychosis Studies, Institute of Psychiatry, Psychology & Neuroscience, King’s College London, London, UK.

^5^ Division of Psychology and Language Sciences, University College London, London, UK.

|  | **Disease-specific GMV reductions** | | **Disease-specific GMV increases** | |
| --- | --- | --- | --- | --- |
| *Disease* | *Coordinates (x y z)* | *Brain region* | *Coordinates (x y z)* | *Brain region* |
| FEP | -24 -22 -23 | Left Parahippocampus | n/s | n/s |
| OCD | n/s |  | -15 -51 -14 | Left Cerebellum |
|  |  |  | 29 -31 -24 | Right Fusiform |
|  |  |  | -27 -1 -20 | Left Amygdala |
|  |  |  | 30 -18 -18 | Right Hippocampus |
|  |  |  | -56 -19 15 | Left Postcentral Gyrus |
| PTSD | n/s |  | n/s |  |
| MDD | 27 -31 -24 | Right Fusiform | 51 -21 4 | Right Temporal Superior |
|  | -21 -48 -18 | Left Cerebellum | -54 -31 7 | Left Temporal Superior |
|  | 30 -18 -17 | Right Hippocampus |  |  |

**Table S1.** Neuroanatomical alterations specific to each disorder. An ANCOVA was used with age, gender and total gray matter volume modelled as covariates of no interest. Neuroanatomical alterations specific to the FEP group were identified by comparing FEP against all other groups (i.e. FEP vs. HC, PTSD, MDD & OCD) and then using the *inclusive* masking option (at p<0.05 uncorrected) in SPM12 software to identify those regions that survived each individual comparison (i.e. FEP vs. HC; FEP vs. PTSD; FEP vs. MDD; FEP vs. OCD). The procedure was followed to identify alterations specific to the other three disorders of interest (PTSD, MDD and OCD). Statistical inferences were made at p<0.05 after family-wise error (FWE) correction with a minimum extent threshold of 10 voxels. Coordinates are reported in MNI space. GMV: gray matter volume; FEP: First Episode Psychosis; OCD: Obsessive Compulsive Disorder; PTSD: Post-Traumatic Stress Disorder; MDD: Major Depressive Disorder; HC: Healthy Controls; n/s: not significant.
